# Degree of Saturation and Free Fatty Acid Content of Fats Determine Dietary Preferences in Laying Hens

**DOI:** 10.3390/ani10122437

**Published:** 2020-12-19

**Authors:** María Palomar, María Dolores Soler, Eugeni Roura, Roser Sala, Olga Piquer, Carlos Garcés-Narro

**Affiliations:** 1 AviFeed Science, Department of Animal Production and Health Public Veterinary Health and Food Science and Technology, Facultad de Veterinaria, Universidad Cardenal Herrera-CEU, CEU Universities, Calle Tirant lo Blanch, 7, E-46115 Alfara del Patriarca, Valencia, Spain; maria.palomarlloris@uchceu.es (M.P.); mariola@uchceu.es (M.D.S.); olga.piquer@uchceu.es (O.P.); 2 Centre for Nutrition and Food Sciences, Queensland Alliance for Agriculture and Food Innovation, The University of Queensland, St. Lucia, QLD 4072, Australia; e.roura@uq.edu.au; 3 Animal Nutrition and Welfare Service, Department of Animal and Food Science, Facultat de Veterinària, Universitat Autònoma de Barcelona, E-08193 Bellaterra, Barcelona, Spain; roser.sala@uab.cat

**Keywords:** fat source, soybean oil, palm oil, laying hen, palatability, double-choice, preference

## Abstract

**Simple Summary:**

Understanding fat sensing in chickens has the potential to improve least cost feed formulation relevant to poultry feeds. Acid oils (soybean acid oil and palm fatty acid distillate) are economical and sustainable feedstuffs with similar fatty acid composition to crude oils (soybean oil and palm oil) but richer in free fatty acids. However, potential issues relevant to the palatability of these oils have been raised. Four experimental diets were offered in a series of double-choice tests to study the effect of free fatty acid content and the unsaturated:saturated ratio on dietary preferences in hens. Hens showed a feed preference for palm oil added diets over soybean oil diets, with palm oil and palm fatty acid distillate being equally preferred. However, the hens demonstrated a preference for soybean oil when offered in choice with soybean acid oil. In conclusion, free fatty acid content and saturation degree affected feed preferences in hens. The use of oils with greater preference values may give rise to greater feed palatability, enhancing feed intake at critical stages.

**Abstract:**

Behavioural and genetic evidence shows that the taste system is intimately related to the sensing of nutrients with consequences for poultry nutrition practices. A better understanding of how chickens may sense fat could provide the background for selecting feedstuffs used in poultry feeds. Acid oils have the potential to be economical and sustainable feedstuffs. These fat by-products from the edible oil refining industry possess a similar fatty acid composition to the crude oils but are richer in free fatty acids (FFA). An experiment was conducted to study the effect of FFA content and the unsaturated:saturated ratio (U:S) on dietary preferences in hens. Four fat sources were added to a basal diet at an inclusion rate of 6%, determining the experimental diets: soybean oil (SO; high U:S, 5% FFA); soybean acid oil (SA; high U:S, 50% FFA); palm oil (PO; low U:S, 5% FFA); and palm fatty acid distillate (PFAD; low U:S, 50% FFA). The experimental diets were offered in a series of double-choice tests to forty-eight Lohmann Brown laying hens housed individually in cages. Each hen was offered the ten potential binary combinations of the four diets including each diet compared to itself (referred to as four control double-choices). Feed intake was measured for two hours twice a day after one hour of fasting. Consumption was analysed as a standard preference index (% of test diet intake in comparison with the total intake). Preference values were compared to the random choice value of 50% using the Student’s *t*-test. None of the four control comparisons differ significantly from 50% (*p* > 0.05), indicating that the changes in preference values observed in the other binary comparisons were related to the dietary changes associated to fat ingredients. Hens showed a feed preference for palm oil added diets over soybean oil diets (*p* < 0.05), with PO and PFAD being equally preferred (*p* < 0.05). However, in this trial the hens demonstrated a preference for SO (low %FFA) when offered in choice with SA (high %FFA) (*p* < 0.05). These results suggest that the degree of saturation plays an important role in dietary fat preferences: hens prefer predominantly saturated oils even when these are rich in FFA. Furthermore, when presented with a choice between predominantly unsaturated oils, hens prefer feed with a low %FFA. In conclusion, %FFA and the U:S ratio affected feed preferences in hens. The use of oils with greater preference values may give rise to greater feed palatability, enhancing feed intake at critical stages.

## 1. Introduction

Taste perception influences feed intake guiding nutrient choices [1,2]. It has been widely assumed that the sense of taste in avian species is not as accurate as in mammals, as they have fewer taste buds [3]. However, the ratio of taste buds to oral cavity volume in birds appears to be higher than in most mammals [4]. Recent behavioural and genomic studies have found that birds have a highly developed sense of taste [1,5,6].

Poultry possess a well-developed capacity to differentiate feed, ingredients and toxic components [7,8]. When choice is limited, animals modulate feed consumption mainly based on energy and amino acid requirements and feed presentation [9,10]. When offered different diets simultaneously, dietary choices in chickens are mainly influenced by potential nutrient deficiencies [11,12]. However, feed preferences may also be influenced by other factors involved in palatability [13,14].

It is generally accepted that there are five basic taste qualities: sweet, sour, umami, bitter and salty. Compared to mammals, chickens have fewer bitter taste receptors and lack the mammalian sweet taste receptor T1R2 [6,15]. In contrast, the ability of chickens to sense fatty acids has been recently uncovered by identifying the involvement of taste receptors FFAR2 and FFAR4 [16,17,18,19,20].

Supplemental dietary fats are widely used to satisfy feed energy requirements, due to their high-energy value. Fat by-products such as acid oils from the edible oil refining industry, represent an economical alternative compared to conventional fats and oils [21]. These by-products are characterized by possessing a high proportion of free fatty acids (FFA) [22] which may affect palatability when added to poultry feeds. Due to the importance of the sense of taste in avian nutrition [4,23], it seems necessary to assess palatability and, if possible, optimize the use of cheaper and environmentally sustainable feedstuffs, improving the profitability of egg production by replacing high-cost ingredients.

Most published studies on chicken dietary preferences assessed long-term feed intake associated to physiological needs [9,11,12,24,25,26]. In contrast, much less is known on short-term feed intake drivers in chickens. Short-term trials assessing palatability are usually performed by double-choice tests, where animals choose between two different feeds offered simultaneously, enabling palatability to be determined [27,28,29,30,31,32].

The present study aimed at documenting laying hen preferences for a range of fat sources differing in FFA content and the degree of saturation (unsaturated:saturated; UFA:SFA) using double-choice tests.

## 2. Materials and Methods

### 2.1. Animals and Housing

All experimental procedures followed European Union guidelines for animal care and handling [33] and were approved by Animal Research Ethics Committee of the Universidad Cardenal Herrera-CEU (CEEA 17/018). The animal study was conducted at the Universidad Cardenal Herrera-CEU Teaching and Research Farm (Náquera, Valencia, Spain).

Forty-eight Lohmann Brown laying hens of 54 weeks of age were selected, possessing a body weight of between 2073 and 2363 g (2221 ± 77 g, mean ± standard deviation). The hens were individually housed in cages (76.2 × 63.0 cm^2^ with a minimum height of 45.0 cm) and animal interaction was avoided by using dividers. The cages were equipped with a nest, perch and three freshwater nipple drinkers. Prior to the experimental trial, a training period (7 days) was implemented to facilitate the hens’ adaptation to the experimental facilities and management.

### 2.2. Experimental Design

Four test diets were evaluated in pairs using a double-choice protocol adapted from Solà-Oriol et al. [29]. Each hen was fed the six possible binary combinations of the experimental feeds offered in two adjacent identical aluminium containers (31.0 × 10.5 × 5.5 cm^3^).

Potential position effects (right vs. left and hen location) were accounted by including as treatment four controls (one for each diet) where the same diet was offered in both feeders. In total, 10 double-choice tests were assessed. The duration of the tests was 10 days. With the objective of reducing the experimental error, a Latin square design was carried out. All potential binary combinations were tested once in each hen, row, column and floor and all were tested every day. The order of the double-choice tests was randomized for each hen.

### 2.3. Diets and Feeding

All experimental diets consisted of a basal diet (94%) and the remaining 6% from one of four dietary oil sources: soybean oil (SO), soybean acid oil (SA), palm oil (PO) or palm fatty acid distillate (PFAD) (Table 1). Oils were kept refrigerated (2 °C for 2 months) prior to mix with the basal diet (spiral automatic feed mixer; Gruber Hermanos, S.A., Spain). Two of the diets (SO and SA) had a high ratio of unsaturated (UFA) to saturated fatty acids (SFA), while the palm-oil-based diets (PO and PFAD) had a low UFA to SFA ratio. Two of the diets (SO and PO) had a low FFA content (5%) while SA and PFAD had a high FFA content (50%). The SO, SA and PO diets had a single oil source while PFAD had an oil blend, consisted of 33% crude palm oil combined with 66% palm FA distillate (and thus possessing the same FFA content as SA). The fatty acid composition of the experimental diets is shown in Table 2. Butylated hydroxytoluene (BHT), a synthetic antioxidant, was incorporated into soybean diets (SO and SA) at 100 ppm. The diets were formulated to cover the nutrient requirements of laying hens in accordance with Fundación Española para el Desarrollo de la Nutrición Animal (FEDNA) recommendations [34] and remained stored in a cool, dry place until the beginning of the test (2 months).

**Figure 1:**
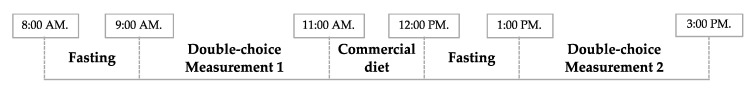
Feeding protocol scheme.

Figure 1 shows the feeding protocol [36]. In brief, after an hour of fasting, two test-containers, each one filled with 100 g of one of the experimental feeds, were fixed side-by-side to the front of each cage. Feed intake was measured for the following 120 min. This procedure was repeated twice daily. In order to avoid bias, the position of the test-containers was switched from one measurement to the next. Between the two measurements, birds were offered a commercial diet for the first 60 min and fasted during the next 60 min before the start of the second test of the day.

The overall feed consumption was calculated as measurement 1 plus measurement 2. During the non-test period the hens were fed with a commercial diet and water was provided ad libitum during all tests. Cages were checked to ensure access to feed and to minimize any possible feed wastage.

### 2.4. Measurements and Calculations

Feed intake was determined by weighing the test-containers before and after each test. The consumption was analysed as a standard preference index (% of test diet intake compared to total intake) [29]. The following mathematical equation was used (1):

Preference (%) = Test diet intake/Total intake × 100(1)

Preference values ranged from 0 to 100% with 50% indicating no preference. Values significantly higher or lower than 50% indicated a preference or aversion, respectively.

### 2.5. Statistical Analysis

Prior to evaluate the preference, the Shapiro–Wilk normality test was performed for each set of preference values. Then, preference values were compared with the 50% no-preference value using the One-sample Student’s *t*-test, for each compared feed. Cohen’s d was calculated after every *t*-test to calculate size effect, with the following code: d = 0.2: small effect; d = 0.5: medium effect; d = 0.8: large effect [37]. Significant differences were considered for *p* < 0.05. The statistical analysis was carried out entirely with IBM SPSS Statistics 24.0 for Windows [38].

## 3. Results

The preference values registered for the 10 double-choice tests are presented in Table 3. All of them resulted in a normal distribution.

As expected, when hens were offered the same diet in both test-containers, the preference measured for control pairs did not differ (*p* > 0.05) from 50% for any of the four experimental diets (SO, SA, PO and PFAD).

The results obtained showed a clear preference for palm diets (PO and PFAD) over soybean diets (SO and SA), irrespective of the %FFA. In particular, PO was significantly (*p* < 0.01) preferred to SO and SA with preference values of 60.88% and 68.98%, respectively. Similarly, PFAD was preferred to SO and SA with values 57.04% (*p* < 0.05) and 72.9% (*p* < 0.01), respectively. PO and PFAD treatments resulted in the greatest preference values and there were no significant differences in palatability between the two (*p* > 0.05), regardless of the degree of acidity of the fat source. However, an effect of the %FFA on preference was observed when comparing SO to SA indicating that hens significantly preferred the low (SO) to the high (SA) FFA content (*p* < 0.01; preference value = 62.1%).

Hens demonstrated a consistent preference for palm-oil formulations over soybean-oil formulations, particularly over SA. Highly significant effect on preference values for PFAD compared to SA (Cohen’s d > 0.8) was observed.

## 4. Discussion

The double-choice test is probably the most widely used method compare palatability values between two diets. Any pair of feeds can be offered to determine which one is preferred by measuring the amount consumed after a given period of time. To study the palatability of more than two diets, it is common to use a reference diet and deduce the relative palatability of feeds that have not been compared directly [29,30]. In this study, four different experimental diets were evaluated in a double-choice protocol without a reference diet: each hen was fed once with each possible two-way combination in a complete block design. Likewise, to rank the four fat sources, it was necessary to make six comparisons.

The specific role of fats, and particularly of acid oils, in palatability has not yet been investigated. It has been reported that chickens have higher intakes when long chain triglycerides are abundant in feed compared to medium chain triglycerides [13,39,40]. Furthermore, Sawamura et al. [19] observed that chickens prefer corn oil (that contains oleic acid and is linoleic acid-rich) rather than mineral oil. The current study showed robust evidence that hens prefer palm oils to soybean oils when given a free choice (*p* < 0.05). Similar results have been observed in pigs [41]. In contrast, a significant preference for SO over PO was reported in a long-term preference trial in laying hens [42]. Understanding the potential effects of the duration of experimental tests (short vs. long term) remains an elusive aspect in feeding choices that deserves additional research.

The aim of our study focused on the short-term reaction of the hens to the feed offered to assess palatability. Previous studies suggest that, if two isoenergetic feeds are compared (one with low and the other with high-fat content), birds will present higher intakes of the high-fat feed, presumably due to better palatability [4,43]. The different fat sources studied were added to a basal diet on a weight basis (6%) and it was decided not to adjust for energy value as this might affect feed palatability.

However, palatability depends not only on the sensory properties of the feed. There are many factors that may affect feed preference in double-choice trials: previous experiences, genetic background, physiological state and environmental and social context [30,44]. In the current study, all the hens were of the same breed and age and had a similar body weight. The birds were placed in individual cages to avoid animal interaction and eliminate errors associated with feeding competition. Furthermore, a training period was implemented prior to the start of the trial, as the absence of a prior learning process might reduce the ability of the hens to make sensible choices [45,46].

Potential preferences for feeder location were also taken into account in the experimental design. Switching container positions between measurements served to minimize the effect of hens favouring a particular position when feeds are clearly differentiated by flavour [8,19,27,47], although some authors maintain that this can confuse the animals and confound the results of the tests [30,48,49].

To validate the trial, control comparisons were included and no differences between the fat sources were observed for any of the controls tested (*p* > 0.05). The double reference control comparisons did not deviate significantly from 50% in any of the possible options (SO vs. SO, SA vs. SA, PO vs. PO and PFAD vs. PFAD). Therefore, any deviation from equal amounts of feed consumed when two types were presented together can be attributed to some desire on the part of the bird [23,28,29].

On another note, the energy density of the diets is a strong driver of intake [10]. However, small differences in energy content do not explain the differences in feed preference. For example, the SO is only 64 kcal/kg more energetic than SA diets (this is <2%) while difference in preference values was very large (62.10% and 37.90%, respectively). Thus, the results presented are consistent with the reports from Solà-Oriol et al. [41] in pigs in that the degree of saturation of fat sources contributes to palatability. Soybean diets have higher polyunsaturated fatty acid (PUFA) (SO = 58.51%; SA = 51.01%) than palm diets (PO = 24.26%; PFAD = 23.01%), which are more susceptible to oxidation than monounsaturated fatty acid (MUFA) and SFA [50]. The rate of FA oxidation increases in relation to their degree of unsaturation. Therefore, predominantly unsaturated oils (i.e., soybean oil) are more unstable than predominantly saturated oils (i.e., palm oil) [51,52,53]. Lipid oxidation produces peroxides which may further break down to form unpleasant flavour and aroma compounds characteristic of what is known as “oxidative rancidity” or “flavour reversion” [53]. 2-Pentyl furan has been identified as a major contributor to flavour reversion of SO [50,54]. On that note, these results are in partial contradiction with previous studies suggesting that unsaturated fatty acids maybe less preferred due to oxidation. FFA are more susceptible to autoxidation than esterified fatty acids and also act as strong pro-oxidants [55,56,57,58]. Although acid oils (having a high %FFA) also have a rancid odour, in this trial no aversion to PFAD was found in hens. Furthermore, we observed a highly significant effect (Cohen’s d > 0.8) between PFAD and SA diets. The lack of significant differences between preference levels for PO and PFAD suggest that high %FFA is acceptable to hens when it comes from a predominantly saturated oil. On the other hand, hens are sensitive to levels of FFA when provided with a choice between SO and SA. This is consistent with the findings of Mistry and Min [56], who reported that FFA decreased the surface tension of SO and increased the diffusion rate of oxygen into the oil, accelerating the oxidation process.

## 5. Conclusions

During diet formulation, the nutritional value of feedstuffs is usually the main criterion for selecting feed composition and palatability is often not considered. Few studies have addressed the effects of the UFA:SFA ratio, rancidity and FFA concentrations on avian taste preference. This study showed that the amount and type of dietary fat sources (%FFA and U:S ratio) affect feed preferences. Hens prefer predominantly saturated oils over unsaturated oils even when they are rich in FFA. However, a clear effect of FFA can be observed in unsaturated oils, with hens preferring feed with a low %FFA. These findings may allow nutritionists to choose oils to better suit laying hen preferences, especially at critical stages when feed intake may be otherwise depressed.

## Figures and Tables

**Table 1:** Composition of the experimental diets.

Ingredients, %	SO	SA	PO	PFAD
Barley 2 rows	49.9	49.9	49.9	49.9
Soybean meal 47.5% Crude Protein	24.3	24.3	24.3	24.3
Corn	6.9	6.9	6.9	6.9
Calcium carbonate (fine-grained)	6.8	6.8	6.8	6.8
Crude soybean oil (SO)	6.0	-	-	-
Acid soybean oil (SA)	-	6.0	-	-
Crude palm oil (PO)	-	-	6.0	1.98
Palm fatty acid distillate (PFAD)	-	-	-	4.02
Calcium carbonate (coarse-grained)	2.1	2.1	2.1	2.1
Sunflower 36% CP	1.9	1.9	1.9	1.9
Monocalcium phosphate	1.1	1.1	1.1	1.1
Sodium chloride	0.3	0.3	0.3	0.3
Methionine (MHA)	0.2	0.2	0.2	0.2
Vitamin and mineral premix ^1^	0.2	0.2	0.2	0.2
Feed sanitizer ^2^	0.2	0.2	0.2	0.2
Sodium bicarbonate	0.1	0.1	0.1	0.1
Choline chloride 75%	0.1	0.1	0.1	0.1
White cereal enzymes	0.05	0.05	0.05	0.05
Red synthetic pigment	0.03	0.03	0.03	0.03
6-phytase 100	0.03	0.03	0.03	0.03
BHT ^3^	0.01	0.01	-	-
**Estimated nutrient composition**				
Gross energy, kcal/kg	3836	3772	3699	3625
Dry matter, %	90.79	90.79	90.79	90.79
Crude protein, %	17.55	17.55	17.55	17.55
Lysine, %	0.914	0.914	0.914	0.914
Methionine, %	0.433	0.433	0.433	0.433
Methionine + Cysteine, %	0.751	0.751	0.751	0.751
Threonine, %	0.657	0.657	0.657	0.657
Tryptophan, %	0.219	0.219	0.219	0.219
Ash, %	12.52	12.52	12.52	12.52
Crude fat, %	7.48	7.48	7.48	7.48
Crude fibre, %	4.16	4.16	4.16	4.16
Calcium, %	3.79	3.79	3.79	3.79
Total Phosphorus, %	0.720	0.720	0.720	0.720
Digestible Phosphorus, %	0.455	0.455	0.455	0.455
Chloride, %	0.225	0.225	0.225	0.225
Sodium, %	0.161	0.161	0.161	0.161
Choline, %	0.031	0.031	0.031	0.031
Xanthophylls, mg/kg	8.344	8.344	8.344	8.344

^1^ Provides per kg of feed: vitamin A, 9000 IU; vitamin D3, 3000 IU; vitamin E, 13 IU; vitamin B1, 1 mg; vitamin B2, 4 mg; vitamin B6, 1.8 mg; vitamin B12, 10 μg; vitamin K3, 1.7 mg; folic acid, 0.3 mg; niacin, 20 mg; pantothenic acid, 8mg; biotin, 52 mg; choline, 200 mg; Fe (from FeSO_4_·7H_2_O), 32 mg; Co (from 2CoCO_3_·3Co(OH)_2_·H2O), 7 mg; Zn (from ZnO), 65 mg; Mn (from MnO), 85 mg; Se (from Na_2_SeO_3_), 0.35 mg; I (from Ca(I_2_O_3_)_2_), 0.7 mg. ^2^ Form-Ad Plus (Adiveter). ^3^ Butylated hydroxytoluene.

**Table 2:** Fatty acid and lipid fraction composition of the diets ^1^. Results expressed as % total fatty acids.

Fatty Acid Composition	SO	SA	PO	PFAD
C12:0	-	-	-	0.41
C14:0	-	-	0.81	0.95
C16:0	11.82	12.70	34.59	36.42
C16:1n7	-	0.21	-	-
C18:0	4.04	3.84	4.00	4.11
C18:1n9	23.21	28.88	34.79	33.70
C18:1n7	1.26	1.08	0.80	0.77
C18:2n6	51.59	48.38	22.76	21.53
C18:3n3	6.92	2.64	1.50	1.48
C20:0	0.41	0.61	0.43	0.41
C20:1n9	0.26	0.28	0.21	0.21
C22:0	0.49	0.93	0.10	-
C24:0	-	0.46	-	-
SFA	16.75	18.55	39.93	42.31
MUFA	23.48	29.36	35.01	33.91
PUFA	58.51	51.01	24.26	23.01
UFA: SFA	4.90	4.33	1.48	1.35
FFA	5	50	5	50

^1^ Fatty acid determination by direct transesterification [35]; fatty acid quantification by gas chromatography (HP 6890 Series II GC System with Flame Ion Detection (FID), Agilent Technologies). SFA: saturated fatty acid; MUFA: monounsaturated fatty acid; PUFA: polyunsaturated fatty acid; UFA: unsaturated fatty acid; FFA: free fatty acid. The symbol “-” indicates a non-quantifiable value.

**Table 3:** Feed preference values for different fat sources offered in a double-choice setup (left diet in every pair is used as the test one and compared with the 50% preference).

Double Choice			N	Preference, %			S.E.M *, %	*p* **	Cohen’s d
SO	vs.	SA	48	62.10	vs.	37.90	3.26	0.001	0.530
SO	vs.	PO	48	39.12	vs.	60.88	3.40	0.002	0.462
SO	vs.	PFAD	48	42.96	vs.	57.04	3.31	0.039	0.310
SA	vs.	PO	48	31.02	vs.	68.98	3.48	<0.001	0.787
SA	vs.	PFAD	48	27.10	vs.	72.90	2.93	<0.001	1.127
PO	vs.	PFAD	48	50.88	vs.	49.12	2.53	0.729	0.050
SO	vs.	SO ***	48	51.86	vs.	48.14	2.44	0.450	0.110
SA	vs.	SA ***	48	48.68	vs.	51.32	2.52	0.603	0.075
PO	vs.	PO ***	48	52.09	vs.	47.91	1.91	0.278	0.158
PFAD	vs.	PFAD ***	48	47.30	vs.	52.70	2.13	0.212	0.182

* S.E.M.: Standard error of the mean. ** *p*: Preference values were compared with the 50% no-effect level. Significance was taken as *p* < 0.05. *** Indicate the four control treatments consisting of double choices between two identical feeds.
